# Community-Based Management of Acute Malnutrition to Reduce Wasting in Urban Informal Settlements of Mumbai, India: A Mixed-Methods Evaluation

**DOI:** 10.9745/GHSP-D-17-00182

**Published:** 2018-03-21

**Authors:** Neena Shah More, Anagha Waingankar, Sudha Ramani, Sheila Chanani, Vanessa D'Souza, Shanti Pantvaidya, Armida Fernandez, Anuja Jayaraman

**Affiliations:** a Society for Nutrition, Education and Health Action, Mumbai, India.

## Abstract

**Background:**

We evaluated an adaptation of a large-scale community-based management of acute malnutrition program run by an NGO with government partnerships, in informal settlements of Mumbai, India. The program aimed to reduce the prevalence of wasting among children under age 3 and covered a population of approximately 300,000.

**Methods:**

This study used a mixed-methods approach including a quasi-experimental design to compare prevalence estimates of wasting in intervention areas with neighboring informal settlements. Cross-sectional data were collected from March through November 2014 for the baseline and October through December 2015 for the endline. Endline data were analyzed using mixed-effects logistic regression models, adjusting for child, maternal, and household characteristics. In addition, we conducted in-depth interviews with 37 stakeholders (13 staff and 24 mothers) who reported on salient features that contributed to successful implementation of the program.

**Results:**

We interviewed 2,578 caregivers at baseline and 3,455 at endline in intervention areas. In comparison areas, we interviewed 2,082 caregivers at baseline and 2,122 at endline. At endline, the prevalence of wasting decreased by 28% (18% to 13%) in intervention areas and by 5% (16.9% to 16%) in comparison areas. Analysis of the endline data indicated that children in intervention areas were significantly less likely to be malnourished (adjusted odds ratio, 0.81; confidence interval, 0.67 to 0.99). Stakeholders identified 4 main features as contributing to the success of the program: (1) tailoring and reinforcement of information provided to caregivers in informal settings, (2) constant field presence of staff, (3) holistic case management of issues beyond immediate malnourishment, and (4) persistence of field staff in persuading reluctant families. Staff capabilities were enhanced through training, stringent monitoring mechanisms, and support from senior staff in tackling difficult cases.

**Conclusion:**

NGO–government partnerships can revitalize existing community-based programs in urban India. Critical to success are processes that include reinforced knowledge-building of caregivers, a high level of field support and encouragement to the community, and constant monitoring and follow-up of cases by all staff levels.

## Abstract (precis)

Under the NGO–government partnership, wasting among children under age 3 decreased by 28% in intervention areas and by only 5% in comparison areas. Success factors included persuading and engaging with communities including delivery of tailored information, close presence and supervision of field staff, and holistic management of other issues beyond acute malnutrition. This intensive approach may be challenging for the government to adapt effectively at large scale.

## INTRODUCTION

Tackling child malnutrition is critical for child survival and sustainable development.^1^^–^^4^ Globally, 17 million children under 5 suffer from severe wasting, and India is home to approximately one-third of these children.^5^^,^^6^ Though recent national surveys show improvements in child malnutrition indicators across India, absolute levels remain high, with 15% of children being wasted or low weight-for-height.^7^^,^^8^

The Ministry of Women and Child Development in India has been running the Integrated Child Development Services (ICDS) since 1975 to address child malnutrition. ICDS tracks the underweight or weight-for-age status of children up to 6 years of age through a network of informal preschools called Anganwadi centers. Each center is staffed with a community-based worker and helper who provide nutritional supplementation to children and pregnant women, basic informal education to children, and health education to mothers. While there are currently around 1.3 million Anganwadi centers functioning across local communities in India, the current policy focus for wasted children has been on hospital-based management through nutrition rehabilitation centers.^9^ Limitations of hospital-based treatment include poor cultural acceptance, hospital-acquired infections, and high relapse rates, which highlight the need for community-based therapeutic care for acute malnutrition in India.^10^^–^^12^

The World Health Organization (WHO) recommends community-based management of acute malnutrition (CMAM), which includes community outreach for the screening of acutely malnourished children, outpatient management, provision of ready-to-use therapeutic food (RUTF), and in-patient treatment of medical complications.^13^ While CMAM has been adopted by many countries worldwide, India has yet to formulate national guidelines for a CMAM strategy.

WHO recommends community-based management of acute malnutrition, but India has yet to formulate national guidelines.

The intervention studied in this article is an adaptation of the CMAM approach that was designed for integration with ICDS growth monitoring activities at the Anganwadi centers. The intervention was implemented by the Society for Nutrition, Education and Health Action (SNEHA), in partnership with ICDS, in urban informal health settlements of Mumbai, India, to reduce the prevalence of wasting among children under age 3. This article reports on a quantitative evaluation of wasting prevalence in intervention communities along with a qualitative study of stakeholders who identified salient features that contributed to the success of the program.

## PROGRAM DESCRIPTION

The SNEHA CMAM program was implemented between November 2011 and March 2016 to provide a continuum of care for pregnant women and children up to age 3 in Dharavi, an informal settlement in Mumbai. The approach incorporated prevention strategies for all children, along with active case-finding and screening for wasting by SNEHA frontline health workers. Children were categorized as experiencing either severe acute malnutrition or moderate acute malnutrition, based on weight-for-height anthropometric measurements. While the program's primary focus was on treatment of children who were wasted, key activities also included monthly growth monitoring of all children at the Anganwadi centers, home-based counseling on infant and child feeding practices to pregnant women and mothers of all infants under 6 months of age, promotion of vaccinations, appropriate referrals to public health facilities, and regular follow-up. Severely wasted and eligible moderately wasted children were also given a locally produced RUTF called medical nutrition therapy. An overview of SNEHA CMAM program activities is provided in Table 1.

**TABLE 1.** Overview of Program Activities

Activity	Description of Activity
Growth monitoring	SNEHA and ICDS frontline health workers jointly mobilized caregivers to bring children to the Anganwadi centers for monthly growth monitoring. The weight and height of all children in the community ages 0 to 6 years was measured. SNEHA frontline health workers used a mobile application to calculate weight-for-height nutrition grades and track information for children under age 3. Monthly growth monitoring enabled SNEHA frontline health workers to screen for severely and moderately wasted children and identify children at risk, including children faltering into malnutrition.
Home visits by SNEHA frontline health workers	SNEHA frontline health workers visited the homes of pregnant women and specific target groups of children under age 3 (severely wasted, moderately wasted, 0–6 months of age, and sick children). Caregivers, typically mothers, were counseled on various topics, including nutritious food habits and choices, age-appropriate nutrition, lactation and weaning, immunization, hygiene, and access to health services. SNEHA frontline health workers were trained on the topics of counseling and effective communication skills. Each target group had a specific protocol for the nature and frequency of visits, and program officers monitored the frequency and quality of the home visits.
Community-based medical nutrition therapy distribution	Medical nutrition therapy is a nutrient-dense dietary supplement for treating severely malnourished children. The peanut- and milk-based preparation was provided by the MCGM NRRC. Severely wasted or severely underweight and wasted children ages 7 to 36 months who have passed an appetite test and had no medical complications were eligible for medical nutrition therapy. Children were screened for complications by a pediatrician at a health camp or the NRRC before medical nutrition therapy treatment was initiated. Children were typically prescribed an 8-week treatment protocol with doorstep delivery of supplements and monitoring of consumption by SNEHA frontline health workers.
Health camps	Periodic health camps were organized by SNEHA in community spaces where a pediatrician checked wasted and sick children referred by SNEHA frontline health workers. Doctors validated the nutrition grade of the child, screened for complications, treated illnesses, prescribed medical nutrition therapy, and referred children for inpatient care. Other children also accessed the camps for minor ailments.
Referrals for MCGM and ICDS health services	Health posts are primary health facilities run by MCGM and responsible for numerous health prevention and promotion activities including distribution of iron-folic acid tablets and vitamin A syrup, deworming drives, immunizations, and detection and treatment of tuberculosis, leprosy, and malaria. SNEHA frontline health workers referred cases of illness and immunization to health posts and facilitated participation in deworming and vitamin A drives. The NRRC health facility is a center for inpatient and outpatient management of children with severe acute malnutrition; the NRRC validated the anthropometry conducted by SNEHA frontline health workers, conducted appetite tests and prescribed medical nutrition therapy, admitted and treated children with minor complications, and further referred children with severe complications to LTMGH, the tertiary MCGM hospital. SNEHA frontline health workers also referred children for services provided by ICDS including cooked meals and take-home rations.
Group meetings and community events	Health behavior change activities were conducted in the community, in partnership with MCGM and ICDS, through games, group discussions, celebrations, cooking demonstrations, and screening of educational movies. Events included breastfeeding initiation ceremonies, baby shower celebrations, and International Breastfeeding and Nutrition week. Every 6 months, mothers whose children recovered and remained recovered from severe wasting were celebrated in the community.

Abbreviations: ICDS, Integrated Child Development Services; LTMGH, Lokmanya Tilak Municipal General Hospital; MCGM, Municipal Corporation of Greater Mumbai; NRRC, Nutrition Research and Rehabilitation Center; SNEHA, Society for Nutrition, Education and Health Action.

Along with ICDS, the SNEHA program worked with the community health posts and the tertiary hospital, Lokmanya Tilak Municipal General Hospital (LTMGH), run by the Municipal Corporation of Greater Mumbai (MCGM). The Nutritional Rehabilitation and Research Center (NRRC), located within the LTMGH Urban Health Center, screens and admits severely wasted children and also provides medical nutrition therapy.

### Intervention Areas

The program covered approximately 300,000 people in Dharavi in 4 phases, encompassing 300 Anganwadi centers. The 300 centers included all those that were functioning in Dharavi at the time of program implementation; ICDS organized the centers into 10 administrative areas, consisting of 30 centers per administrative area. From inception, the SNEHA CMAM program screened over 30,000 children under age 3 and over 5,600 pregnant women.

The SNEHA CMAM program screened over 30,000 children under age 3 and over 5,600 pregnant women.

### Intervention Team

**FIGURE 1.**
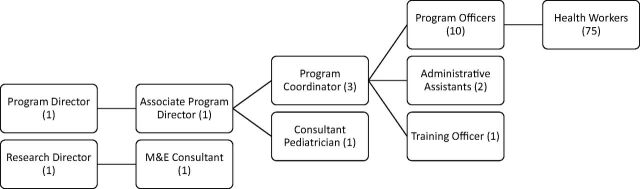
Organogram of SNEHA CMAM Program, January–March 2016 Abbreviations: CMAM, Community-based Management of Acute Malnutrition; M&E, monitoring and evaluation; SNEHA, Society for Nutrition, Education and Health Action.

The program relied on a broad base of frontline health workers who delivered program activities. At the time of the study, approximately 75 frontline health workers and 10 program officers supervised frontline health workers at the field level, all employed by SNEHA. Three program coordinators led the field teams and reported to the associate program director and program director. The program had a full-time training officer who was assisted by the program coordinators in organizing and leading the trainings. External technical experts were also brought in to assist and co-lead trainings. Figure 1 depicts the staffing structure of the SNEHA CMAM program.

### Profile and Training of Frontline Health Workers

Each frontline health worker collaborated with 3 Anganwadi centers to cover all pregnant women and children under 3 in the service areas, covering at least 750 households. Each frontline health worker monitored approximately 150 to 180 children at a given time during the intervention. Frontline health workers received intensive and repeated trainings, with emphasis on building both knowledge and skills. Box 1 provides an overview of frontline health workers in the program.

BOX 1 **Frontline Health Workers in the Society for Nutrition, Education and Health Action (SNEHA) Community-based Management of Acute Malnutrition (CMAM) Program**

**Background:** Close to 90% of the frontline health workers were women and over two-thirds were married. Approximately 78% had completed secondary school (tenth grade), and over 50% of all frontline health workers had completed higher secondary school (twelfth grade).

**Recruitment and remuneration:** Frontline health workers were recruited through advertisements or through informal networks, followed by interviews with senior program staff. They were paid a fixed monthly allowance and their contracts were renewed annually. Routine performance-based incentives were not given; however, during community events their hard work was appreciated and verbally praised.

**Responsibilities:**

- Understand community needs and develop rapport with community members.
- Conduct home visits with target groups (malnourished children, pregnant women, and children under 6 months of age).
- Work with Integrated Child Development Services (ICDS) frontline health workers to conduct anthropometry of all children under 6 years of age.
- Ongoing identification of children under 3 and pregnant women.
- Plan, mobilize attendees, facilitate, and document community group sessions on relevant topics.
- Assist with events, campaign logistics, and mobilizing community participation.
- Refer cases to municipal health facilities and follow up on cases.

**Supervision:** Program officers supervised and randomly observed frontline health workers conducting home visits. They provided individualized feedback on the quality of the counseling, covering both communication strategies and technical content. Frontline health workers reported all activities completed to their program officers on a daily basis. Home visits, anthropometry sessions, and event details were recorded manually and electronically in smartphones through the CommCare mobile application.

**Training:** Frontline health workers are intensively trained for knowledge and counseling skills. Details of the different types of training given to them are illustrated below.

**Training Details for Frontline Health Workers: Illustrative Data From 2015**

In 2015, 30 new training sessions, lasting 6 hours each, and 18 follow-up/repeat sessions, lasting 2–4 hours each, were conducted. Of the 30 new training sessions, 11 were thematic, 2 covered technical issues, 13 focused on skills development, and 4 centered on behavioral issues.

- **Thematic:** Sessions that impart knowledge on breastfeeding, anthropometry, the importance of nutrition, localized recipes, household and personal hygiene, illness management, antenatal and prenatal care, and child development.
- **Technical:** Sessions that cover use of the CommCare mobile application.
- **Skills development:** Sessions that cover interpersonal communication, art of listening, negotiating with the community, and body language in the community.
- **Behavioral skills:** Sessions that enable field health workers to cope with their own emotions while working in the community and to maintain relationships in the community.

### Program Objectives

The primary objective of the SNEHA CMAM program was to reduce the prevalence of wasting among children under 3 years old residing in the intervention areas of Dharavi. Secondary objectives included improved infant and young child feeding practices, immunization coverage, vitamin A supplementation, and utilization of government health services.

### Program Funding and Costs

The SNEHA CMAM program raised funds through philanthropic foundations, corporate foundations, and individual philanthropists. The funders had no role in study design, data collection, data analysis, or preparation of this article. The total approximate running cost of the program covering the entire duration of the program was 56.5 million Indian rupees (INR), or approximately US$868,217 (Reserve Bank of India Reference Exchange Rate, US$1 = INR 64.5, July 2017). The main costs were for project personnel (64%) and project administration and implementation (33%), which included training, meeting and event costs, monitoring and evaluation costs, and other field expenses. The cost of running the program was to some extent subsidized because of the already available network of municipal health facilities, including an established and well-run NRRC at the local municipal hospital. Costs were also minimized through the use of lower-cost, locally produced RUTF provided by the NRRC.

**Figure:**
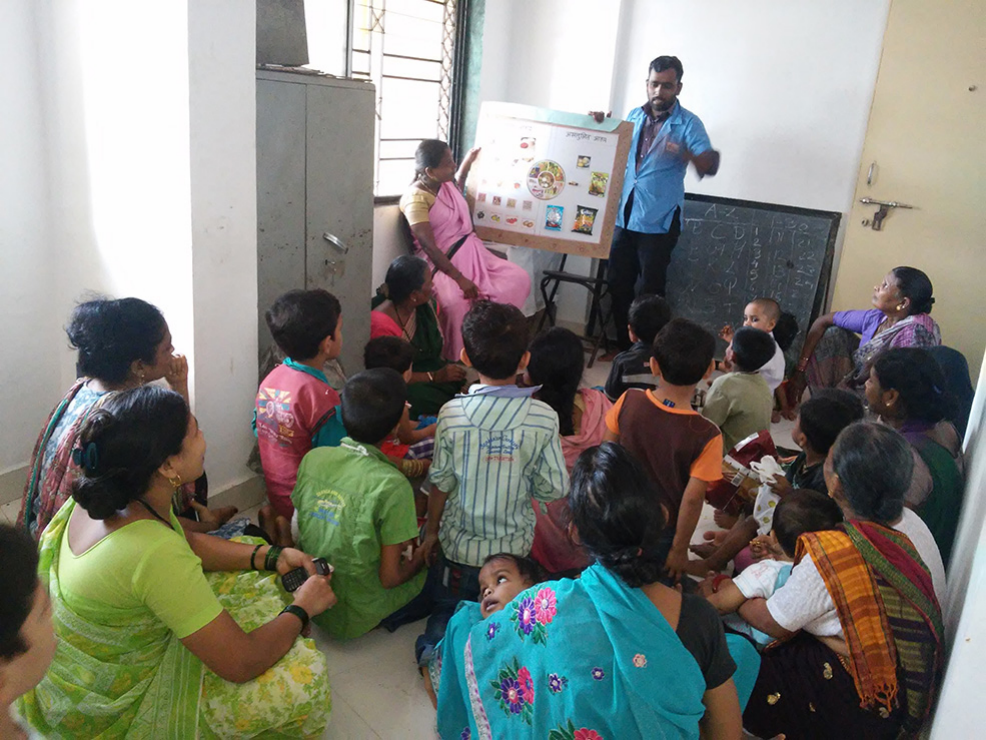
Frontline health workers from the Society for Nutrition, Education and Health Action and Integrated Child Development Services conduct a group session together. © 2015 Aahar field team/SNEHA

### Routine Program Monitoring

The software used for electronic data collection, CommCare, is an open-source, mobile-based platform to aid community health workers in data collection and case management. The application functions offline where Internet connectivity is limited and enabled frontline health workers to continuously track and update the status of women and children that were screened. Questionnaires to monitor key outcomes and processes during routine activities were finalized using paper-based forms and then programmed in the CommCare application. The information collected by frontline health workers was sent to the server over a cellular data network and downloaded to Microsoft Excel for reporting and analysis. The system can be easily developed by project staff and scaled throughout an organization or government entity committed to the process of electronic data collection and standardized reporting. Please refer to the supplement for more details of the program.

## METHODS

We used a quasi-experimental cross-sectional survey design with a comparison area to examine prevalence and association of wasting with program exposure among children under 3 in intervention areas. We also conducted a qualitative study between January and July 2016 across all 10 areas of intervention, including pilot areas.

### Quasi-Experimental Study Design

#### Selection of Intervention Areas

Due to the complex nature of the intervention, SNEHA piloted various iterations of the program processes across the first 150 Anganwadi centers (5 administrative areas) between 2011 and 2013, and then scaled up the program to the remaining 150 centers in 2014. To study the effectiveness of the SNEHA CMAM approach, we evaluated the 5 administrative areas where the CMAM program was implemented after piloting was completed.^14^ All 150 Anganwadi centers from the 5 scale-up areas were included in the quasi-experimental evaluation and the SNEHA CMAM program ran for approximately 16 months before endline data collection.

In intervention areas, we collected baseline information from March through July 2014 from the caregivers of children under age 3. Intervention area baseline questionnaires covered socioeconomic status (education, occupation, asset ownership, housing status), household and environmental sanitary conditions (water supply and treatment, toilet ownership), infant and young child feeding practices, illness prevalence in children (diarrhea and acute respiratory infections), and utilization of government services for maternal and child health (ICDS, MCGM). We included the 2012 Progress out of Poverty Index (PPI) to assess the likelihood that a household is living below specific poverty lines. The PPI incorporates household characteristics (number of children residing in household, type of fuel, father's education, and occupation) and asset ownership (10 small and large assets). Height/length and weight of the youngest child under 3 and the height and weight of the mother were taken.

#### Selection of Comparison Areas

In consultation with ICDS, we purposively selected 107 Anganwadi centers in nearby informal settlements of Wadala, Mumbai, as the comparison area. To the best of our knowledge, no nutrition rehabilitation centers were available in those areas, nor was there any other organization other than ICDS providing child nutrition services, such as growth monitoring, counseling, or food supplementation to children under age 3. The objective of the baseline in comparison areas was to give us a reliable indication of whether the areas were plausible comparison areas to implement a more rigorous evaluation at endline. We conducted a short baseline survey with the primary caregivers of children under 3, between September and November 2014, to measure the prevalence of wasting, the 10 indicators that encompass the PPI to compare socioeconomic characteristics, and any exposure to SNEHA activities.

#### Seasonality

Seasonality and associated periods of food insecurity have been linked to higher rates of malnutrition in rural India.^15^ In our experience, the incidence of wasting in urban informal settlements of our intervention areas were similar over an entire year, with the exception of the heavier monsoon months of July and August when children suffer greater levels of illness and diarrhea. We did our best to avoid collecting data during this heavy monsoon period. We examined our monitoring intervention data to estimate monthly incidence of wasting, as well as the monthly prevalence estimates of wasting from the evaluation data. We did not observe any clear patterns of seasonality that would impact our results.

The incidence of wasting in urban settlements remained constant, with the exception of July and August during heavy monsoons.

The questionnaire used at baseline in intervention areas was also used at endline, from October through December 2015, with additional questions on program processes and migration patterns during the intervention period. The same endline survey tool was used in both intervention and comparison areas; investigators collected all information electronically using the CommCare mobile application.

#### Sample Size

We estimated sample sizes to capture a 25% reduction in wasting prevalence from baseline to endline in intervention areas. We assumed 15% prevalence of wasting at baseline, rho=.04, alpha=.05, 2-sided, and power=.80. At baseline we sampled caregivers in all 150 intervention service areas, with a target of 18 respondents per area, totaling a target sample of 2,700 interviews. At endline we sampled each center in the intervention area with a target of 23 respondents, totaling a target sample of 3,450 interviews.

We increased the sample size for the intervention areas at endline due to our experience with the fluctuating nature of residency in the informal settlements. Throughout the intervention we observed substantial mobility of children and families in and out of the communities. Families went to the villages where they were born for extended periods, moved within the same informal settlement, moved to other parts of Mumbai, or they left the city altogether. In our monitoring data we estimated that approximately 30% of the children who left our program before turning 3 years of age fit in this broad category of children who were no longer at the residence where they lived at the time of screening. Assuming the influx of families was similar to the observed outflow, we increased the intervention-area target samples at endline in case we wanted to limit the intervention sample to children with a minimum level of time-exposure to the intervention areas. In the comparison areas, we sampled 107 Anganwadi center service areas at baseline and at endline, with a target of 20 respondents per center, approximately 2,140 total respondents for each round.

#### Data Collection Procedures

We used a modified systematic sampling approach to recruit respondents in the intervention areas. We conducted a house listing process, followed by random selection of a household as the starting point. Investigators spun a pen to determine which direction to move and then continued to every third household within the boundaries of that Anganwadi center service area until the target number of caregivers were identified and agreed to be interviewed. If a caregiver had more than one child under age 3, information was collected for the youngest child.

In the comparison areas, where we had not conducted a house listing activity due to resource constraints, the boundaries within each center service area were shown to the investigators and their supervisors by the ICDS staff. These endpoints were the starting points for data collection and investigators continued on to every third household within the service area until the target sample size was achieved.

Anthropometric data was collected using a standardized protocol. Bubble levels (manufactured by Freemans Measures Pvt. Ltd.) were used to verify that weighing locations were flat. Children and infants under 2 years of age lay or sat on an electronic baby weighing scale (manufactured by Nitiraj Engineers Pvt. Ltd.) with an accuracy of +/− 10 grams and their lengths were measured on an infantometer (manufactured by Meditrin Instruments Pvt. Ltd.). All children over 2 years of age were weighed on an adult mechanical weighing scale and their heights were measured using a measuring tape with an accuracy of +/− 0.1 cm and a wooden triangle to assist in marking the height with a pencil. Investigators confirmed that the scale was at 0 before the weighing of each child, and weighing scales were calibrated daily. Investigators took all measurements twice in pairs, with random cross-checking by supervisors. Indicators of wasting are derived from standardized *z* scores of weight-for-height/length anthropometric measurements. Wasting is defined as a weight-for-height *z* score 2 standard deviations below the median WHO growth standard (<−2 SD) and severe wasting is 3 standard deviations below the median WHO growth standards (<−3 SD). Anthropometric *z* scores were calculated using Emergency Nutrition Assessment software. Children with outlier values of weight-for-height *z* score >5 SD or <−5 SD were removed from the analysis, along with children who had significant discrepancies in their height or weight measurements.^16^

#### Statistical Analysis

All statistical analyses were done in STATA 12.0 (StataCorp, College Station, TX). To compare prevalence levels of wasting, socioeconomic characteristics, infant and young child feeding practices, immunization, and government program coverage outcomes across intervention and comparison areas, we used Pearson chi-square for categorical variables and *t* tests for means. For the regression analysis of the endline data, we created a dummy variable for children living in the intervention or comparison areas. We estimated mixed-effects logistic regression models where the outcome of wasting was regressed against the intervention dummy variable, adjusting for child, maternal, and household characteristics selected from literature.^17^^–^^20^

### Qualitative Study Design

We undertook a qualitative study between January and July 2016 across all 10 intervention areas, including pilot areas. The primary source of data for this study was in-depth interviews with 37 stakeholders (24 mothers and 13 staff).

#### Interviews With Mothers

We purposively sampled 24 mothers from all 10 administrative areas using the following criteria: their children were severely wasted, moderately wasted, or normal; few complicated cases that needed hospital intervention; severely wasted children who consumed/did not consume medical nutrition therapy; families that needed intensive persuasion to participate; children who relapsed; and women who were pregnant. Data from the interviews were triangulated with information from 3 focus group discussions with frontline health workers, 12 observation sessions, a review of monitoring data, and analysis of purposively selected success case stories routinely documented by frontline health workers. One limitation of case stories was that they documented information related to program successes only. Approximately 140 case stories were available from 2015; we chose 46 diverse case studies.

#### Staff Interviews

All senior staff implementing the program were interviewed. Three of 10 program officers and 6 of 75 frontline health workers were purposively selected for the interviews to obtain diversity in age, gender, and length of association with the program. The majority of the staff were female. Staff who had been associated with the program for less than 6 months were excluded.

A team of 2 independent investigators conducted the interviews and the focus group discussions. The duration of the interviews was 40 minutes on average, with a range of 20 to 90 minutes. The team interviewed mothers in their homes in Hindi and program staff in SNEHA field offices in a mixture of Hindi and English. We translated the tools for the study from English to Hindi and pretested them. We dropped 1 interview from the analysis due to non-completion. A summary of data collection methods used for the qualitative study is provided in Table 2.

**TABLE 2.** Data Collection Methods for the Qualitative Study

Category	Details	Themes Explored
**In-depth interviews (N=37)**		
Senior staff	Program head (discussion), implementation management (n=3), health camp doctor (n=1)	General experiences with the program, its conceptualization and its components, changes in the program, perceived achievements and challenges, working in partnerships, and sustainability under different models of implementation. Health camp doctor: implementation of the camp, perceived benefits of the camp, challenges, and role of health camps in the program.
Field team	SNEHA frontline health workers (n=6), SNEHA program officers (n=3)	Sharing typical work-week activities and roles played, experiences, perceived achievements and challenges faced, and case illustrations.
Community	Total interviews: 24 Maximum diversity sample including mothers of severely wasted, moderately wasted, non-wasted, pregnant women; by age (0–6 years and above)/gender/religion; purposively sampled some complicated cases)	Stories of interaction and familiarity with the program, perspectives (positive and negative) on various activities, suggestions for the program.
**Focus group discussions (N=3)**		
Field team (SNEHA frontline health workers)	3 focus group discussions, with 6 SNEHA frontline health workers per group	Protocols, time allocation to different activities, interaction with the community, program officers, and senior management.
**Observations (N=12 sessions)**		
Home visits by SNEHA frontline health workers	Over 2-week period: 8 visits, approximately 20 minutes each	General processes, nuances of interaction with the community, process through which the SNEHA frontline health workers communicate information.
Growth monitoring	Over 2-week period: 4 sessions, half a day each	Growth monitoring process in the Anganwadi centers, community mobilization, and management of crowd during weighing and taking of measurements.
Site visit	NRRC and urban health post, 1-time observation	Crowd, physical infrastructure, placement of SNEHA staff at the NRRC, general familiarity of the field staff with the place.
**Descriptive monitoring data and case stories**		
Descriptive monitoring data	Already exists in the program	Size and coverage of the program—number of beneficiaries, number of children monitored, number of home visits recorded.
Case stories	Already existed in the program to document successful cases to promote best practices. About 140 stories were recorded in 2015; we selected 46 diverse cases, translated them into English, and analyzed them.	Interaction of the field team with caregivers, process of identification of malnutrition, intervention with the family.

Abbreviation: SNEHA, Society for Nutrition, Education and Health Action.

#### Management and Analyses of Qualitative Data

All interviews and focus group discussions were voice-recorded, transcribed verbatim into English, and anonymized. We entered the transcripts and field notes from observation in NVivo (version 10). We analyzed the data using the 3 steps described by Miles and Huberman: (1) data reduction (selecting, simplifying, and condensing data systematically), (2) data display (organizing information in structured models or themes), and (3) drawing conclusions (through careful examination of the displays generated).^21^ We used generic thematic analyses techniques for the data reduction process, wherein data was sifted through and codes were affixed to blocks of text. The preliminary themes used to examine program activities were decided before conducting interviews (see Table 2 for the list of themes). After preliminary analysis, we open-coded for the following: (1) broad ideas that cut across different implementation components and were perceived by the community and staff to contribute to program success, and (2) mechanisms through which the program worked in the community. Two rounds of formal discussions were held, wherein all authors gave input on the findings.

### Ethical Approval

The protocol, questionnaires, and informed consent forms for the CMAM program evaluation were reviewed and approved by the Institutional Ethics Committee, Holy Family Hospital, and Medical Research Centre, Mumbai. All respondents gave their written informed consent before participation in quantitative surveys and qualitative interviews and focus group discussions. Additionally, for the qualitative interviews, we recorded consent verbally when respondents from the community were not literate. All women we approached for the qualitative assessment consented to participate in the data collection.

## RESULTS

### Quasi-Experimental Study

For the baseline, a total of 2,578 caregivers were interviewed in intervention areas and 2,092 caregivers were interviewed in comparison areas. At endline, a total of 3,455 caregivers were interviewed in intervention areas and 2,122 caregivers in comparison areas. Table 3 provides demographics of households in intervention and comparison areas at endline. Demographics among children (e.g., age, gender, and birth weight) in both areas were similar at endline. Although mothers were similarly educated and largely not working, the mothers' mean body mass index (BMI) and mean age at marriage were significantly lower in the comparison areas at endline. Mean BMI was 22.8 in intervention areas and 22.3 in comparison areas (*P*≤.01); similarly, mean age at marriage was 20.3 years versus 19.9 years (*P*≤.05). Mothers in intervention areas had a significantly different distribution in the location of their natal homes than mothers in comparison areas. Households in comparison areas had a significantly higher likelihood of poverty than intervention areas (73.7% versus 71.4%). However, households in intervention areas reported a significantly higher level of food insecurity in terms of worrying whether the household would have enough food to eat (21.7% as compared with 18.1%).

**TABLE 3.** Household Demographics in Intervention and Comparison Areas at Endline, October–December 2015

	Intervention (N=3,455)^a^	Comparison (N=2,122)^a^
**Children**		
Age, months, mean	16.3	16.8
Female, %	46.3	47.6
Low birth weight (<2.5 kg), %	21.5	21.3
**Mothers**		
Education, %		
Illiterate, primary, informal	20.1	21.2
Secondary (grades 5–10)	56.7	57.7
Higher secondary and above (grade 11 and above)	23.2	21.0
Not employed, %	93.8	94.9
Body mass index, mean	22.8	22.3**
Place of birth, %		
South India	7.7	2.4***
North and Central India	17.5	29.2
East and Northeast India	12.0	5.1
West India	14.5	25.9
Mumbai	48.4	37.4
Age at marriage, mean	20.3	19.9*
**Households**		
Years of residence in Mumbai, %		
Less than 1 year	1.5	1.6
1–5 years	6.4	5.0
6 or more years	92.1	93.3
Treatment of drinking water,^b^ %	32.9	32.1
PPI: Likelihood below US$2.16/day/PPP line, %	71.4	73.7**
Private toilet, %	20.4	15.8
Food insecurity,^c^ %	21.7	18.1*
Religion, %		
Muslim	45.2	37.5
Hindu	49.9	57.1
Other	5.0	5.5

Abbreviations: PPI, Progress out of Poverty Index; PPP, purchasing power parity.

Pearson chi-square for categorical variables and *t* tests for means.

* *P*≤.05; ** *P*≤.01; *** *P*≤.001.

a Total sample sizes may vary due to missing values generated from data cleaning.

b Treatment of drinking water includes chlorine, use of filter, solar disinfection, and boiling.

c Question: *“In the last month did you worry that your household would not have enough food?”*

At baseline, caregivers in both comparison and intervention areas reported negligible levels of services received by SNEHA at less than 2%. At endline, after 16 months, more than 85% of sampled caregivers in intervention areas reported that they received services from SNEHA for their youngest child. Less than 1% of sampled caregivers in the comparison areas reported any services from SNEHA at endline, indicating that contamination was negligible. At endline we also asked caregivers whether the child had received any services from other organizations in the previous month; in both intervention and comparison areas, less than 1% reported getting any service from another organization for that child (data not shown). At both baseline and endline, PPI poverty likelihood estimates were significantly higher in the comparison areas, but socioeconomic levels remained relatively constant in both comparison and intervention areas.

At baseline, overall prevalence of wasting was not significantly differently between intervention and comparison areas. Prevalence of wasting from baseline to endline decreased by 28% (18.0% to 13.0%) in intervention areas and by 5% (16.9% to 16.0%) in comparison areas (Table 4). Severe wasting prevalence fell by 39% from baseline to endline—from 3.8% to 2.3%. The differences in overall wasting and severe wasting prevalence levels at endline between intervention and comparison areas were significant, whereas the moderate wasting prevalence in intervention areas was lower but not significant.

**TABLE 4.** Wasting, Socioeconomic Status, and Coverage of Services in Intervention and Comparison Areas at Baseline (March–July 2014 for Intervention, September–November 2014 for Comparison) and Endline (October–December 2015)

	Baseline		Endline	
	Intervention (N=2,578)^a^	Comparison (N=2,092)^a^	Intervention (N=3,455)^a^	Comparison (N=2,122)^a^
Wasting, %	18.0	16.9	13.0	16.0**
Severe wasting, %	3.8	3.3	2.3	3.9**
Moderate wasting, %	14.2	13.6	10.6	12.1
PPI: Likelihood below US$2.16/day/PPP line, %	72.6	77.3***	71.4	73.7**
Child received any service from SNEHA in previous month, %	2.2	1.6	86.0	0.8***

Abbreviations: PPI, Progress out of Poverty Index; PPP, purchasing power parity; SNEHA, Society for Nutrition, Education and Health Action.

Pearson chi-square tests comparing baseline intervention to baseline comparison areas and endline intervention to endline comparison areas.

* *P*≤.05; ** *P*≤.01; *** *P*≤.001.

a Total sample sizes may vary due to missing values.

We evaluated the program through logistic regression analysis of the endline data, comparing children residing in intervention areas with children residing in the comparison areas. Three logistic regressions models were used for the outcome of wasting: Model 1 adjusted for child characteristics; Model 2 adjusted for child and maternal characteristics; and Model 3 adjusted for child, maternal, and household characteristics (Table 5). All 3 models showed that children residing in intervention areas had significantly lower odds of being wasted (adjusted odds ratio [AOR], 0.81; 95% confidence interval [CI], 0.67–0.99 in Model 3), compared with neighboring comparison areas. Other factors for children that were also significantly associated with lower odds of wasting in all models included: being female (AOR, 0.76; 95% CI, 0.64–0.90 in Model 3); having a higher birth weight (AOR, 0.57; 95% CI, 0.49–0.66 in Model 3); having a mother with secondary level education as compared with primary or no education (AOR, 0.78; 95% CI, 0.63–0.98 in Model 3); and having a mother with a higher BMI (AOR, 0.96; 95% CI, 0.94–0.98 in Model 3). Factors for children significantly associated with a higher odds of wasting in all models included: having a mother born in east and northeast India as compared with south India (AOR 1.61; 95% CI, 1.00–2.59 in Model 3); a mother with higher age at marriage (AOR, 1.03; 95% CI, 1.00–1.06 in Model 3); having a household with a higher PPI poverty likelihood (AOR, 2.06; 95% CI, 1.13–3.74 in Model 3); and being Hindu as compared with being Muslim (AOR, 1.23; 95% CI, 1.01–1.49 in Model 3).

**TABLE 5.** Multi-Level Logistic Regression Analysis of Household Demographics Associated With Wasting at Endline, October–December 2015

	Null Model (N=5,524)	Unadjusted OR (N=5,524)	Model 1^a^: AOR (95% CI) (N=4,913)	Model 2^a^: AOR (95% CI) (N=4,889)	Model 3^a^: AOR (95% CI) (N=4,869)
β° (SE)	0.154 (0.008)	0.180 (0.013)	1.122 (0.261)	1.551 (0.798)	0.673 (0.463)
**Children**					
Resides in intervention area		**0.78 (0.64, 0.94)** **	**0.80 (0.66, 0.97)** *	**0.81 (0.67, 0.98)** *	**0.81 (0.67, 0.99)** *
Age, months			1.00 (0.99, 1.01)	1.00 (0.99, 1.01)	1.00 (0.99, 1.01)
Female			**0.75 (0.63, 0.89)** **	**0.75 (0.64, 0.89)** **	**0.76 (0.64, 0.90)** **
Birth weight			**0.53 (0.46, 0.62)** ***	**0.56 (0.48, 0.65)** ***	**0.57 (0.49, 0.66)** ***
**Mothers**					
Education					
Illiterate, primary, informal				1	1
Secondary (grades 5–10)				**0.75 (0.60, 0.93)** **	**0.78 (0.63, 0.98)** *
Higher secondary (grade 11 and above)				**0.74 (0.57, 0.97)** *	0.82 (0.62, 1.09)
Not employed				1.05 (0.67, 1.66)	1.04 (0.66, 1.64)
Body mass index, mean				**0.96 (0.94, 0.98)** ***	**0.96 (0.94, 0.98)** ***
Place of birth					
South India				1	1
North and Central India				1.34 (0.87, 2.06)	1.34 (0.86, 2.07)
East and Northeast India				**1.60 (1.00, 2.54)** *	**1.61 (1.00, 2.59)** *
West India				1.23 (0.80, 1.89)	1.21 (0.78, 1.87)
Mumbai				1.22 (0.82, 1.83)	1.29 (0.86, 1.95)
Age at marriage, mean				1.02 (0.995, 1.05)	**1.03 (1.00, 1.06)** *
**Households**					
Years of residence in Mumbai					
Less than 1 year					1
1–5 years					1.11 (0.52, 2.33)
6 or more years					0.92 (0.47, 1.81)
Treatment of drinking water^b^					0.89 (0.74, 1.08)
PPI: Likelihood below the US$2.16/day/PPP line					**2.06 (1.13, 3.74)** *
Private toilet					0.89 (0.70, 1.14)
Food insecurity^c^					1.09 (0.88, 1.35)
Religion					
Muslim					1
Hindu					**1.23 (1.01, 1.49)** *
Other					1.12 (0.73, 1.73)
SD (SE)	0.433 (0.061)	0.416 (0.061)	0.356 (0.072)	0.330 (0.077)	0.325 (0.078)
Intracluster correlation coefficient	0.05	0.05	0.04	0.03	0.03

Abbreviations: PPI, Progress out of Poverty Index; PPP, purchasing power parity; SD, standard deviation; SE, standard error.

Statistical significance is calculated using mixed-effects logistic regression models.

* *P*≤.05; ** *P*≤.01; *** *P*≤.001.

a Model 1 adjusted for child characteristics; Model 2 adjusted for child and maternal characteristics; and Model 2 adjusted for child, maternal, and household characteristics.

b Treatment of drinking water includes chlorine, use of filter, solar disinfection, and boiling.

c Question: “In the last month did you worry that your household would not have enough food?”

Children in intervention areas had significantly lower odds of being wasted than children in comparison areas.

Analysis of secondary outcomes, including infant and young child feeding practices and immunization levels, in comparison and intervention areas showed mixed results (Table 6). Exclusive breastfeeding prevalence improved significantly in intervention areas (from 48.6% to 66.6%) from baseline to endline, but the difference between intervention and comparison areas at endline was not significant. Comparing baseline and endline results in intervention areas showed no significant improvements in prevalence estimates of timely initiation of breastfeeding, continued breastfeeding, timely complementary feeding, introduction of solid foods, and children being fully immunized. There were significant positive differences from baseline to endline and between intervention and comparison areas for dietary diversity (26.9% to 35.0% from baseline to endline and 4.5 percentage points higher than comparison areas), consumption of iron-rich foods (29.6% to 40.1% from baseline to endline and 9.5 percentage points higher than comparison areas), and vitamin A supplementation (73.7% to 81.2% from baseline to endline and almost 10 percentage points higher than comparison areas). However, consumption of foods rich in vitamin A was significantly lower in intervention areas (24.8% to 20.5% from baseline to endline and 7.3 percentage points lower than comparison areas). Utilization of health services provided through government partners for the youngest child under age 3 also improved significantly in intervention areas. For ICDS services, utilization increased from 29.0% to 60.7% from baseline to endline, approximately 31 percentage points higher than in comparison areas. For MCGM services, utilization increased from 35.4% to 51.5%, approximately 18 percentage points higher than in comparison areas at endline.

**TABLE 6.** Secondary Outcomes in Intervention Areas and Comparison Areas at Baseline (March–July 2014) and Endline (October–December 2015)

	Baseline		Endline			
	Intervention Areas		Intervention Areas		Comparison Areas	
	N	%	N	%	N	%
Timely initiation of breastfeeding (0–23 months)	1899	37.0	2417	37.2	1424	49.5***
Exclusive breastfeeding (<6 months)	488	48.6	679	66.6***	385	66.0
Continued breastfeeding (12–15 months)	315	74.0	456	79.2	314	83.1
Timely complementary feeding (6–9 months)	398	48.0	408	51.5	252	57.5
Introduction of solid foods (6–8 months)	293	51.5	304	53.3	181	56.9
Minimum dietary diversity (6–23 months)	1502	26.9	1863	35.0***	1115	30.5*
Consumed vitamin A-rich foods (6–23 months)	1502	24.8	1863	20.5*	1115	27.8***
Consumed iron-rich foods (6–23 months)	1502	29.6	1863	40.1***	1115	30.6***
Fully immunized (9–23 months)	1208	79.3	1557	81.5	934	72.2***
Received at least 1 vitamin A supplement (9–23 months)	1208	73.7	1557	81.2***	934	71.3***
Child received any service from ICDS in previous month, %	2578	29.0	3455	60.7***	2122	29.6***
Child received any service from MCGM in previous month, %	2578	35.4	3455	51.5***	2122	33.4***

Abbreviations: ICDS, Integrated Child Development Services; MCGM, Municipal Corporation of Greater Mumbai.

Pearson chi-square tests comparing baseline intervention to endline intervention and baseline intervention to endline comparison areas.

* *P*≤.05; ** *P*≤.01; *** *P*≤.001.

Analysis of secondary outcomes showed mixed results, with some improvements in child feeding practices but not across all indicators.

Utilization of health services provided through government partners for the youngest child under age 3 improved significantly.

### Qualitative Study

A brief summary of participant demographics for the qualitative study is presented in Table 7.

**TABLE 7.** Demographics of Participants in Qualitative Study

**Staff Interviews**	
Number	13
Age, years, mean (SD)	35.2 (9)
Female, %	62.0
Years of association with the program, mean (SD)	2.7 (1.2)
**Focus Group Discussions With SNEHA Frontline Health Workers**	
Number in each focus group discussion	5 to 6
Age, mean (SD)	32.5 (8.5)
Female, %	81
Years of association with the program, mean (SD)	2.4 (1.2)
**Community Interviews**	
Total number	24
Age of mother, mean (SD) *(n=20)*	26.6 (4)
No. of children in the family, mean (SD) *(n=21)*	3 (2)
Religion, %	
Hindu	54
Muslim	38
Christian	8
Cases severely wasted or moderately wasted, %	63
Cases with medical complications, %	17
Pregnant women, %	17
**Case Stories**	
Number	46
No. of children in the family, mean (SD) *(n=39)*	3 (1)
**Type of Cases**	
Non-wasted children, %	21
Severely wasted/moderately wasted, %	54
Pregnant, %	9
Complicated cases requiring holistic intervention, %	11
Others (contraception, family planning), %	4

Abbreviations: SD, standard deviation; SNEHA, Society for Nutrition, Education and Health Action.

#### General Perceptions on the CMAM Program and Its Activities

The SNEHA frontline health workers were mostly recognized for motivating caregivers to attend growth monitoring activities and for conducting home-based counseling. The community strongly identified the program with the growth monitoring activity; community members reported gathering for the growth monitoring activity when they saw their frontline health worker carrying a black bag with the weighing equipment. Table 8 summarizes the strengths and challenges of key program activities as reported by stakeholders.

**TABLE 8.** Strengths and Challenges of Key Program Activities as Reported by Stakeholders

Strengths	Challenges
**Growth monitoring**	
Growth monitoring had become a regular and well-planned activity at the Anganwadi center. Most mothers acknowledged the usefulness of growth monitoring. Mothers conceded their inability to remember growth monitoring dates; hence, frontline health workers' repeated mobilization of the community was useful.	Most mothers were willing to bring their children for growth monitoring. Resistance to growth monitoring in the community mainly stemmed from practical difficulties (time, work pressure, and migration), rather than issues of cultural acceptance.
**Home visits**	
Home visits by frontline health workers were well-accepted and welcomed by the community. Frontline health workers had been well-trained technically. In addition, most had been trained in and had acquired the soft skills for approaching households as well as for tailoring information. There was considerable oversight of frontline health workers that also played a role in ensuring home visits happened regularly and appropriately.	Some severely wasted children required more visits than those required as per protocol; the frontline health workers often did not record why and when these additional visits were done in the monitoring software. There was a need for training frontline health workers on information pertaining to the entire household rather than focusing on mothers alone.
**Health camps and referrals to NRRC**	
Health camps were held regularly. The community perceived health camps to be useful, mainly due tothe easy access to free medicines and tonics. Field staff felt that the main use of health camps was in confirming whether children were anthropometrically wasted or not. The partnership with NRRC and the adjoining government hospital worked well for the program. The community often reported that frontline health workers referred them to the government hospitaland even accompanied them there if required.	Health camps, when established, were meant specifically for wasted children and pregnant mothers. But it was difficult for camps to turn away other sick children; hence, the camps were largely being used as general health camps for all children, which made them crowded.
**Provision of medical nutrition therapy**	
The logistics for supply and distribution of medical nutrition therapyin the program had been clearly set by the time of scale-up of the program. A checklist format had been developed for tracking medical nutrition therapy consumption of each child; these checklists were being monitored closely.	Consumption of medical nutrition therapy in the program was lower than expected. It was therefore difficult to make strong conclusions on the effectiveness of medical nutrition therapy in this context. Overseeing compliance of medical nutrition therapy consumption by frontline health workers was challenging. Frontline health workers delivered several days of cups at a time to a child, but consumption by the child was self-reported by the mother. While there were no serious issues with logistics (supply and storage) of medical nutrition therapy, there were mothers who found it difficult to feed the medical nutrition therapy cups to the severely wasted child for the full course of 56 days. Mothers and frontline health workers noted that some children got bored of the sweet flavor of the medical nutrition therapy and refused to eat it after a few days. There have been cases of children being pulled out of therapy due to persistent diarrhea or mere refusal to consume.

Abbreviation: NRRC, Nutritional Rehabilitation and Research Center.

A typical home visit by a frontline health worker lasted approximately 15 to 20 minutes, depending on the caregiver's time and availability. Some respondents also mentioned that frontline health workers helped them with referrals to health care services (public and private), including accompanying the mothers to hospitals if needed. Few respondents mentioned group activities conducted by SNEHA. When asked about these activities, mothers often mentioned the difficulties of gathering in a group or finding time to participate in the events. The few participants (3 of 24) who had attended at least 1 group meeting acknowledged that they were useful in providing information. Consumption of medical nutrition therapy in the program was low. A summary of monthly averages for process indicators, including consumption of medical nutrition therapy, is presented in Table 9.

**TABLE 9.** Process Indicators in Intervention Areas (150 Anganwadi Centers)

Activity for Child Under Age 3	Monthly Average Oct 2014–Sep 2015
Total children monitored	7009
Total moderately wasted in the program	617
Total severely wasted in the program	112
Children weighed each month	4834
Moderately wasted children receiving home visits	443
Severely wasted children receiving home visits	89
Children attending health camps	289
Children consuming medical nutritional therapy	24

Source of data: Routine monitoring data of the program.

Consumption of medical nutrition therapy in the program was low.

#### Program Success Factors

Program features that both the community and field staff perceived as critical to its success were:

- **Constant presence of field staff:** The majority of community respondents mentioned the constant presence of program staff in the field as a critical component of the program. The rental of community rooms for field teams to convene and eat lunch ensured that frontline health workers remained in the intervention areas for the duration of the work day. Secondly, the mothers felt the field teams were always accessible because mobile numbers were shared between the staff and the mothers.
- **Information-sharing with the community through reinforcement and as a tailored process:** Repeated information-sharing by program field staff was perceived by the community as one of the most useful features of the program. Most counseling occurred at the individual level and frontline health workers carefully tailored communication. Frontline health workers reported being trained on ways to adapt information to the needs of individual mothers by using appropriate language and through observation of the household: its members (size, education, decision makers, support system for the mother), physical characteristics of the house (hygiene, kitchen, work place), and the mother's receptivity to information.
- **Persistence of field staff and collective persuasion:** A referral system existed within the program to persuade families who were reluctant to use services. Frontline health workers sought help from senior SNEHA and ICDS staff at all levels to persuade families to use appropriate services. In cases of domestic violence or lack of childcare, assistance was sought from other nonprofit organizations.
- **Holistic case management:** Case management of children in the program was not limited to addressing wasting. Along with the management of routine illnesses associated with malnutrition, the SNEHA field staff assisted with emergencies and enabled access to treatment for complicated underlying medical conditions in children (e.g., vision correction or cleft palate surgery). In addition, field staff identified and supported children and mothers whose household environments were detrimental to their well-being, including situations of violence, mental illness, and lack of family support. Anecdotal evidence suggests that children from such households go through repeated bouts of severe malnutrition with intermittent periods of recovery. While these cases are atypical, the community appeared to have a high regard for the CMAM staff because of their intervention in such cases (see illustrative quotes from participants in Table 10).
- **Training and supervision of the program team:** Staff emphasized that training, supervision, and support given to field staff were critical to success. Between June 2014 and March 2016, 74 training sessions were held for program staff on various themes (see Box 1 for illustrative details). Frontline health workers noted 3 aspects of the training that were especially appreciated: (1) each theoretical session was followed by mock practice sessions in the community to practice what they learned, (2) repeated trainings helped refresh their memory and correct misperceptions, and (3) the focus on skill-based training along with knowledge training was useful in their work. Frontline health workers felt that skill-based training taught them how to communicate with mothers in a language and method that mothers would appreciate, assess the mental state of the family they were dealing with, know when to back off and when to continue persuading the family, cope with their own emotions while dealing with difficult cases, and reconcile unsuccessful cases.

**TABLE 10.** Quotes from Participants Illustrating Program Features Contributing to its Success

Themes	Illustrative Quotes
Constant field presence of staff	“Now if the community does not see me for one day, next day I get a call—where are you? Sometimes the community people even directly come to our center to inquire where I am. (Male field staff, 27 years, employed with SNEHA: 1.5 years) “If anyone requires, we give our mobile number so that they can call us when they need any guidance or help. I had given my mobile number to her so she can call me if she has any problem—even in the night. She does not have a proper family support system.” (Female field staff, 34 years, employed with SNEHA: 2 years) “They are here only. They keep coming. They had visited here yesterday only; they gathered several women to explain to them … actually there was a meeting.” (Mother, 30 years, housewife, Muslim, 6 children, youngest girl, age 2 years, was severely wasted, recovered) “They mostly come quite often in a week, like they were here 2–3 days ago. They ask how we are. (Pregnant woman, 28 years, housewife, Christian, 2 kids)
Information-sharing with the community through reinforcement and as a tailored process	“… but doctors telling is different, their telling is different. Doctors are always in a hurry. They are under pressure because of patients, so they tell in shortcuts, some of it I understand and some I don't. These people (from SNEHA) are free, they tell us freely, each and everything, that you do this way. Then they come next day and ask whether we did the way we were told. Then we tell them that yes, we did. Then they again ask us after 1 week whether it was beneficial or not. It happens like this. And what will the doctors say, they just tell, whether we do it not, only we and our children are responsible. This is how doctors do it. And these people come and ask us regularly, ask us again after 1 week about whatever happened, whether the child is eating or not, whether the child is liking it or not, they ask us all of these. (Mother, 24 years, Muslim, 4 kids, 2 younger kids were severely wasted and 1 was on medical nutrition therapy) “SNEHA believes in giving messages, and individualization of messages was very important. So when you do home visits, there are 2 or 3 things which would help. Firstly, it was like you know a message for a particular person only, secondly you come to know the home situation also because there are times when the home situations are interfering with the actual process.” (Senior staff, other details masked to protect identity) “Today in the morning I visit one house. I found some bad smell was coming. So I will not tell immediately. First I will observe how that woman is. I will see the cleanliness in the house. While talking with her we see all how is kitchen maintained.” (Female field staff, 40 years, employed with SNEHA: more than 3 years)
Persistence of field staff and collective persuasion	“When she asked about the registration at that time she told that no, her husband has no time to pick up her to hospital. So she asked her father's mobile number. But they don't have that also. Then SNEHA frontline health worker asked for the neighbor's mobile number. The lady said okay. Then the next day, the SNEHA frontline health worker called her neighbor and spoke to her husband: ‘what is the reason why you did not register her for a pregnancy checkup?’ Then the program officer also called her husband and explained about the importance of the registration, medicine, everything. The next day the SNEHA frontline health worker, the ICDS frontline health worker, and the health post frontline health worker all went to her house and explained jointly. Then her husband took her to the hospital and did the registration. The continuous visits helped the family.” (Case study of a pregnant woman, age not known, Hindu, 3 children) “One family was not ready for immunization. Not even at a private clinic, since one of their relatives died after immunization, they said. The SNEHA frontline health worker spoke to the mother again and again. She agreed but her mother-in-law did not. We all went several times—me, the program officer, ICDS frontline health worker, and even our doctor visited to tell them. We all went together and told them. Then they agreed. (Female field staff, 35 years, employed with SNEHA more than 3 years)
Holistic case management	“When the SNEHA frontline health worker first identified the child, he was 3 months old. She oriented the mother regarding SNEHA and its work. The child had a cleft palate. The SNEHA frontline health worker spoke to the mother regarding her feelings for the child, ongoing treatment, and her difficulties faced while feeding the child. The mother replied that they had recently shifted to Mumbai as her husband worked here and mainly for the child's treatment. She did not know any hospital and was looking for one. While talking, she was upset and in tears. She said that all her relatives blame her for her child's condition and they say that he looks like a mouse. The SNEHA frontline health worker counseled her that it was a birth defect and can be successfully treated with surgery. She referred her to the hospital and screened the child. The mother fed breast milk to the child with a bowl and spoon. Sometimes the mother did get irritated, too, she shared, and felt bad and angry when other people came home to see the child and gave suggestions. When the mother shared her concerns, the SNEHA frontline health worker could feel her helplessness. The SNEHA frontline health worker asked the mother to calm down and said she understood her feelings. The SNEHA frontline health worker inquired about the father and the mother replied that he was nice, but due to family pressure, he also felt it was the mother's fault. The mother cried a lot. In the hospital, the doctors advised an operation. After the operation, the child could feed better. The mother started top feeds and the child gained weight. (Case story of an 8-month-old boy, cleft palate, migrant population, mother's age not known.) “The woman was mentally disturbed. She had a 3-month-old girl. She could not feed the child breast milk and so it was on top feed. The SNEHA frontline health worker visited her house many times and told her not to take stress. The woman said she had a problem with her family and her in-laws; and they were not accepting her. She wailed loudly—‘I am suffering because my husband is not accepting me,’ and she used abusive language. Our concern was that her small daughter will suffer because of this. This case was referred to the prevention of violence against women and children in SNEHA. The program counseled the entire family repeatedly. Now her family has understood and given her permission to live separately with her husband. (Case story of a new mother, 23 years, with a 3-month-old baby girl)

Abbreviations: ICDS, Integrated Child Development Services; SNEHA, Society for Nutrition, Education and Health Action.

The constant presence and accessibility of staff in the field was critical to the success of the program.

**FIGURE 2.**
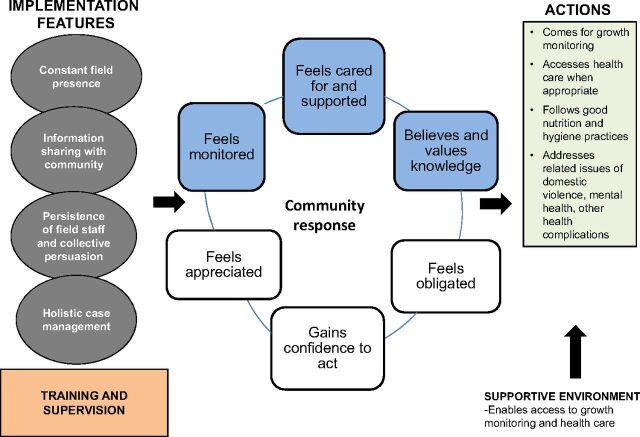
Success Factors of the SNEHA CMAM Program Abbreviations: CMAM, Community-based Management of Acute Malnutrition; SNEHA, Society for Nutrition, Education and Health Action.

The staff also cited the stringent supervision mechanisms as instrumental for ensuring coverage and quality. Frontline health workers were continuously monitored by program officers in the community who always knew their whereabouts. Daily debriefing was done to review and respond to problems as necessary, enabling frontline health workers to get immediate assistance on difficult cases. When we explored whether such tight monitoring mechanisms had a negative influence on the staff, a frontline health worker pointed out that field staff sometimes escalated issues unnecessarily to catch the attention of senior staff; overall, most found the close supervision helpful. See Figure 2 for a chart showing successful program features, as well as the community's response to them and resulting actions.

#### Community Mechanisms in the Program

Mothers reported that they were motivated to participate in program activities due to a combination of the following factors:

- **They felt cared for and supported:** The constant field presence and accessibility of field staff was both acknowledged and appreciated by the mothers. Mothers reported that frontline health workers frequently inquired about their children's well-being, called them for ongoing growth monitoring activities, repeated information for them, and took time to encourage the entire family. The active involvement of senior staff, who also visited the community to persuade reluctant caregivers, sent a clear message to the community that the program staff cared for them.
- **They believed the knowledge imparted by the frontline health workers was useful:** Mothers found the frontline health workers to be a reliable health resource. They felt that the frontline health workers advised them appropriately on diet and feeding practices for their children and pregnant women. Mothers cited useful advice provided by frontline health workers on different types of food, recipes, cooking methods, and the importance of not eating junk food. They also valued information on hygiene, such as strategies for keeping their houses and children clean, and on the importance of immunizations, breastfeeding, and growth monitoring. Mothers expressed that the informal nature of interactions and frontline health workers' patience in explaining things to them gave them a higher degree of comfort than what they experienced with health professionals in hospitals. They often asked the frontline health workers for information on vaccination dates, qualifications of doctors, availability of hospitals in the vicinity, and even on issues such as education for the child, livelihood options, and vocational courses. There was a general consensus among mothers that following the frontline health workers' advice was beneficial for the well-being of their children.
- **They felt monitored:** Mothers acknowledged their tendency to forget the information given by frontline health workers due to their preoccupation with household chores and other activities. The constant presence of the frontline health workers in the community and frequent interaction served as a monitoring mechanism to ensure that the mothers complied with the advice given to them. According to the mothers, the frontline health workers played the role of counselor and overseer by persevering to inculcate healthy practices through reminders and reinforcement of messages, and also by arriving at the house at unexpected moments to inquire on what their children had been fed. This role was both appreciated and desired by the mothers.

**Figure:**
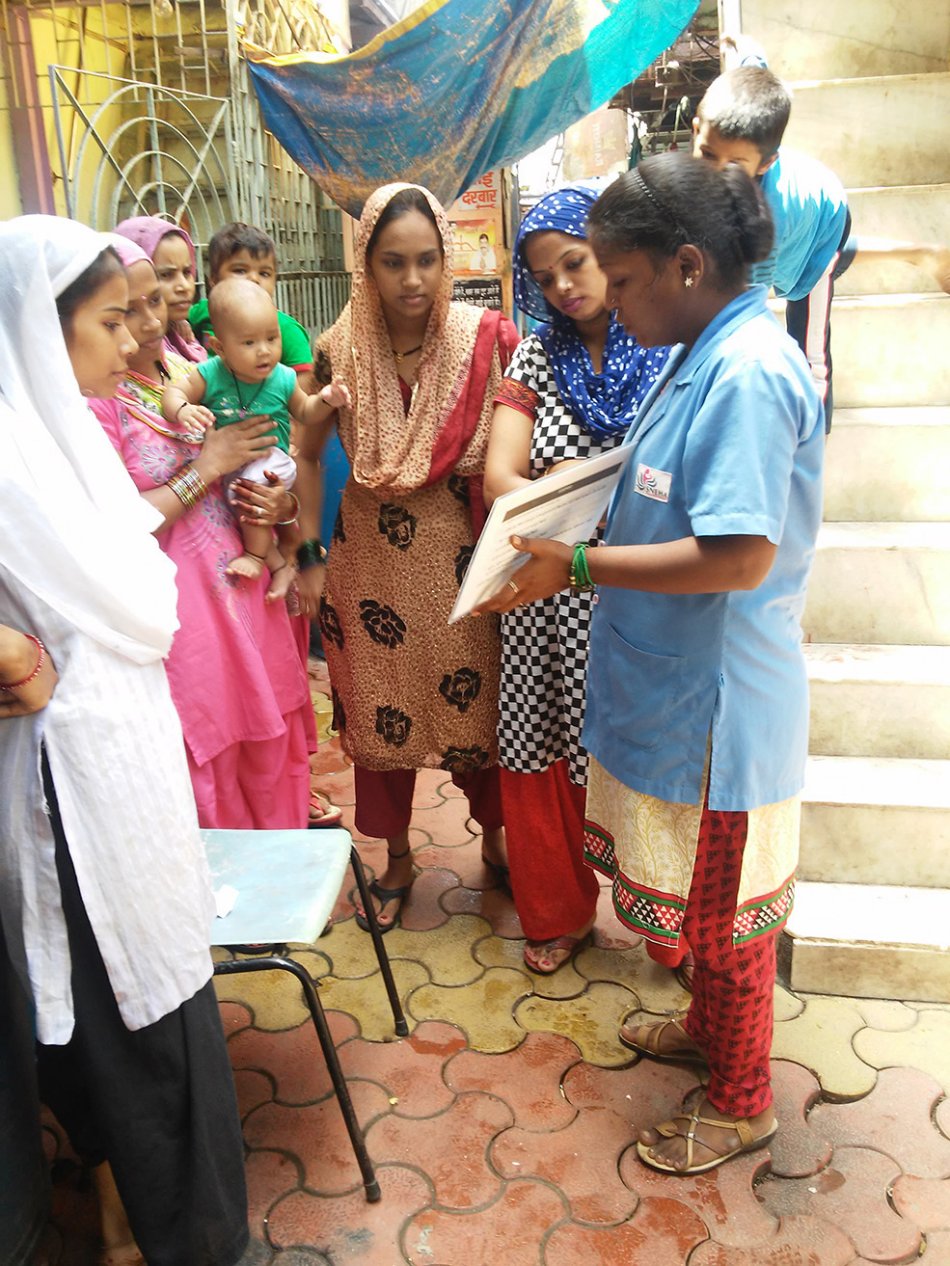
Frontline health workers were constantly present in the community, repeatedly sharing information. © Aahar field team/SNEHA

Three additional themes emerged, although not as strongly: (1) Mothers felt a sense of obligation toward the frontline health workers for spending so much time seeking them out. This made the community feel grateful and motivated them to participate in program activities. (2) Mothers gained confidence to act on child health issues, for example to visit a health facility, since they were armed with both knowledge and support from the SNEHA team. (3) Mothers were encouraged by the appreciation and verbal praise they regularly received from the health workers (see illustrative quotes about successful community mechanisms in Table 11).

**TABLE 11.** Quotes from Participants Illustrating Successful Community Mechanisms in the Program

Themes	Illustrative quotes
Community felt cared for and supported	“I remember how often we had to take [child's name] to the hospital, even in the rains. Now he is okay. Everyone in the neighborhood says that he's come back from death's jaw. There was no hope. His chest was full. [SNEHA frontline health worker] was here at that time, she walked in the rain and first took us to the small hospital, and then walked to the big hospital with us. She stayed until the admission process was over and then she left.” (Mother, 36 years, housewife, Muslim, 6 children, youngest boy, age 1.5 years, was severely wasted with pneumonia complications, recovered) “She [SNEHA frontline health worker] even took me along on 2–3 occasions to the hospital … because I wouldn't understand anything, hence she came along. There she accompanied me for 2 days, then I understood everything. Before I could get there, she would have the case paper ready and would show me the medicines … after that I'd return and she would stay back for some work.” (Mother, 25 years, housewife, Hindu, 2 children, younger girl, age 2.5 years, is severely wasted) “When they are not there, we have to manage on our own. When they [SNEHA frontline health workers] came, we felt a sense of support.” (Mother, 30 years, housewife, Muslim, 6 children, youngest girl, age 2 years, was severely wasted, recovered)
Community believed that the knowledge imparted by the SNEHA frontline health workers was useful	“This person from SNEHA, she comes daily … doctor comes once or 2 times a month … they advise us on weight and tell us about doctors. We get to know if child is not the right weight. We receive information so their visits are beneficial to us.” (Pregnant women, 25 years, housewife, Hindu, 1 girl, age 2 years, severely wasted, recovered) “They ask you to take care of the child, give them milk on time, feed them milk for 15 minutes on one side and then other … that the child should be fed milk 10–11 times through the day, and only then his weight will increase. Children should not be given any food from outside and you should start after 6 months. They should be given all the medicines on time.” (Mother, age not known, housewife, Hindu, 1 girl, age 3 months old, non-wasted)
Community felt monitored	“If they are there, it is good because then parents look after their children properly. They keep coming, so we also have to be attentive to our children.” (Mother, 30 years, housewife, Hindu, 2 children, younger boy, age 4 years, was severely wasted with complications, recovered) “If there is something that you may have forgotten to follow, you will instantly remember it after seeing them. Yes, because I don't feed him properly then how will he grow? That is why as soon as he wakes up in the morning I wash his hands and mouth and then give him milk and thereafter I give him something to eat. By that time what if someone comes to ask me what I fed him? That is why I feed him properly. We will also become careless … because we are being told [by the SNEHA frontline health worker] all the time, so we are attentive and we also fear that they might come anytime to ask us. Because of their visits we would know that today we are supposed to go to check the weight; otherwise, amidst these kids one tends to forget these things. It feels good because they come and call us.” (Mother, 25 years, housewife, Muslim, 4 children, twins were 11 months, girls, one of them was moderately wasted with complications of not walking)

Abbreviations: CMAM, community-based management of malnutrition; SNEHA, Society for Nutrition, Education and Health Action.

## DISCUSSION

The results demonstrate that the SNEHA CMAM program was successful in achieving high levels of coverage and lower levels of wasting, particularly severe wasting, in the program intervention areas. The large decline in severe wasting prevalence was not surprising because the program focused on identifying and treating severely wasted children. By the end of the program, children residing in intervention areas had significantly lower odds of being wasted—by 19%—than in neighboring comparison areas. While most characteristics (birth weight, gender, mother's BMI) significantly associated with wasting in our approach are known, in urban CMAM programs the regional origin of mothers may be another factor to consider in targeting and developing implementation activities.

The secondary outcomes showed mixed results, with some improvements in child feeding practices but not across all indicators. The intervention improved the utilization of health services provided by government partners in the program for children under age 3. These findings are consistent with an evaluation of an NGO–government partnership to enhance ICDS services in rural northern India, where women residing in the intervention area had improved program coverage and breastfeeding practices, but with limited effects on complementary feeding practices.^22^

**Figure:**
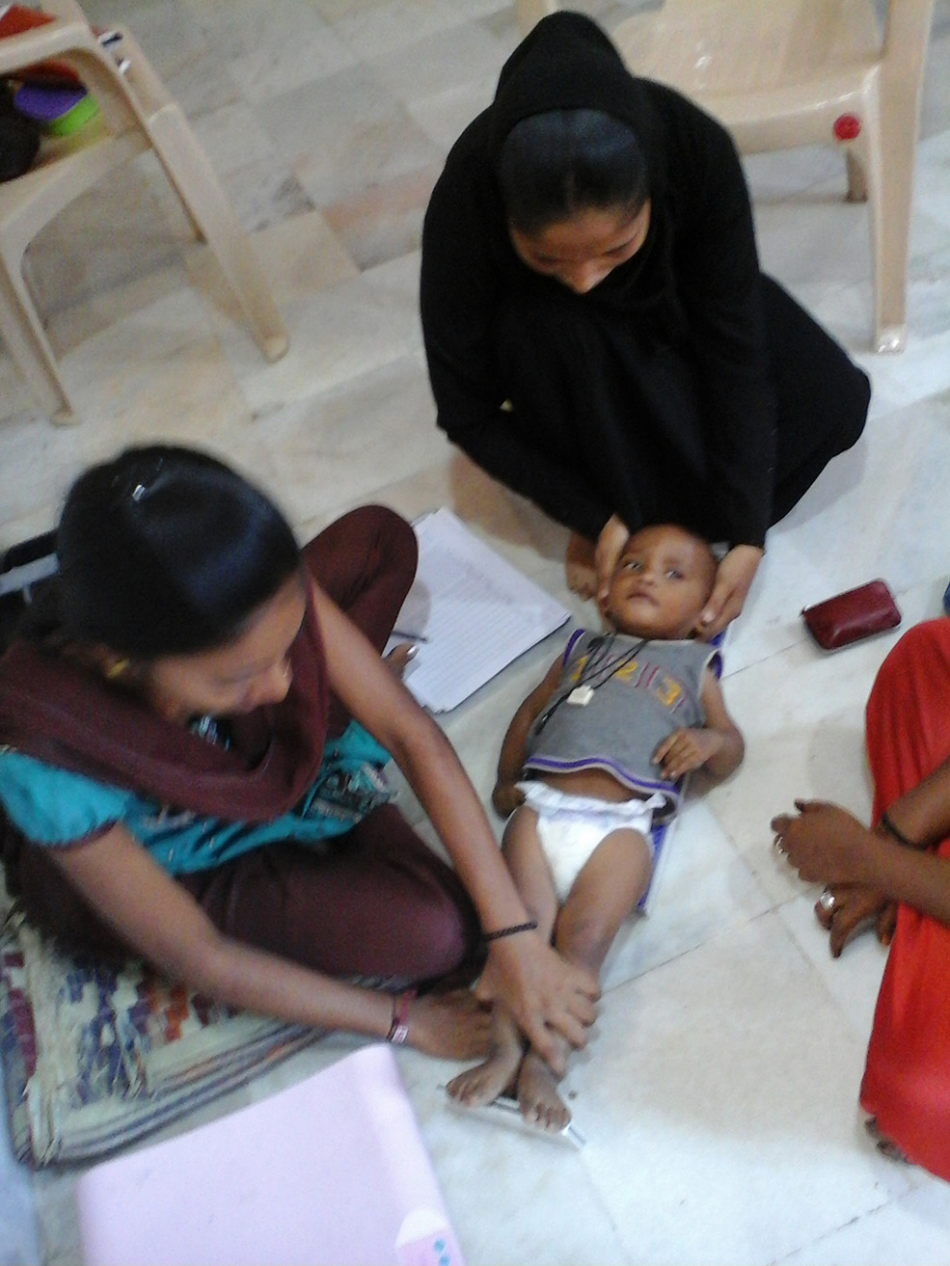
Growth monitoring of an infant conducted by a frontline health worker. © Aahar field team/SNEHA

The core strength of the SNEHA CMAM approach was its intensive and persistent engagement with the community. This need for engagement with the community as a key factor in ensuring success of community-based malnutrition programs has been well-documented in the literature^23^^–^^25^ and was a planned feature of the SNEHA CMAM program. The use of RUTF is currently a subject of debate in academic and practitioner's circles in India, and studies on cultural acceptability and adherence to these regimens in this country are lacking. Unlike CMAM programs in other contexts, overall compliance with the RUTF component by caregivers of severely wasted children in our program was low. This could be attributed to implementation challenges, as well as issues related to community acceptance. From the program point of view, we faced initial logistical issues with supply and storage of medical nutrition therapy. Even with a high level of community engagement and doorstep delivery of medical nutrition therapy cups by the frontline health workers, overseeing the delivery and compliance of medical nutrition therapy consumption in the community setting was a challenge. Retrospectively, the implementation team felt that the promotion of medical nutrition therapy required more intensive programmatic efforts. In addition to implementation difficulties, the repetitive flavor may have contributed to some difficulty in caregivers consistently feeding medical nutrition therapy cups to the severely wasted child for the full course of 56 days. We suggest that other CMAM programs should develop strategies to overcome these issues in the program design phase itself. Box 2 summarizes the key findings from our program.

BOX 2 **Key Findings From the Society for Nutrition, Education and Health Action (SNEHA) Community-based Management of Acute Malnutrition (CMAM) Program**

By the end of the program, children residing in the intervention areas experienced lower levels of wasting than in comparison areas. Successful components of the program included regular growth monitoring of children, intensive home visits, and referrals to locally available primary care. The consumption of ready-to-use therapeutic food in our program was low, possibly due to implementation challenges as well as issues related to community acceptance. We recommend that other CMAM programs develop strategies to overcome these issues during the design phase itself.

Robust community engagement mechanisms can be influential in reducing severe malnutrition among children. For robust community engagement, frontline health workers who are motivated, trained, and well supervised are essential. This can be achieved through appreciation and verbal praise of frontline health workers and repeated training. Training should focus not just on technical aspects of the program, but also on community engagement skills. Supervision includes constant monitoring of frontline health workers, especially while handling difficult cases, random field-level quality checks, and electronic data collection mechanisms for overseeing monthly targets.

To examine the feasibility of a similar programmatic approach to reduce severe malnutrition, programs may consider the following questions:

1. Can pockets of the population be selected for intensive intervention?
2. Are geographical distances amenable to community outreach by frontline health workers and their supervision?
3. Can frontline health workers be trained and motivated? This program recruited a set of in-house frontline health workers who worked closely with the government frontline health workers. In government programs, where long-term frontline health workers are used to set patterns of working, adoption of new skills and routines could be more challenging.
4. Is government support available, in terms of access to local hospitals or provision of facilities, for carrying out the growth monitoring activities?

Specific contextual factors were advantageous to the program. The ICDS Anganwadi centers and health posts were close to the community, and the health posts provided accessible immunizations. The biggest advantage was that houses were spaced closely, which enabled frontline health workers to make frequent home visits to specific households while also monitoring children across the community more generally. The rigorous data monitoring component, which was critical for intensive supervision, was achieved through the use of electronic data collection. After monitoring formats and reporting needs had been established during the pilot phases, they were easily scaled up across the program.

The bulk of program costs were related to employment, training, and supervision of frontline health workers (details are available in the supplement). The cost of running the program was to some extent subsidized by the available network of municipal health facilities, including an established and well-run NRRC at the local municipal hospital (LTMGH). Costs were also minimized through the use of lower-cost locally produced RUTF provided by the NRRC. Adopting components of the SNEHA CMAM program may not be prohibitively costly if the same municipal government infrastructure that supported our program can be integrated with the work of ICDS. While monetary costs of additional training and supervision of ICDS field staff may be feasible, other aspects of implementation need to be considered carefully. The adoption of new skills and new tasks by frontline health workers of programs like ICDS that have a history of poor implementation could be challenging. Partnerships need to be established at various levels between the ICDS staff and the health sector.

A major drawback of the program was the lack of focus on sustainability—that is, handing over responsibility of key program activities to either the government partners or to the communities. In the next phase of the program, having now established credibility for the urban CMAM approach in the communities and with the partners, SNEHA is piloting an approach with the aim of gradually transferring ownership of the program to ICDS. In April 2016, SNEHA has signed a memorandum of understanding with the ICDS Commissionerate and the Mumbai Municipality (MCGM) to formalize the partnership through the Mumbai Child Health and Nutrition Committee. The memorandum of understanding will enable SNEHA, ICDS, and MCGM to coordinate resources, manpower, and sharing of data to serve Mumbai's most vulnerable areas.

Since April 2016, SNEHA frontline health workers in this program no longer participate in direct implementation, but instead they work more intensively to strengthen the capacity of public health systems and ICDS staff in order to build ownership of key processes. This is to be done by increasing ICDS field presence, improving referrals of pregnant women and children to the public health system, and thereby increase access and utilization of public health facilities and ICDS services. The program is working toward strengthening the capacity of not just the ICDS frontline health workers, but also their supervisors. After experiencing the success of the partnership working with SNEHA frontline health workers, we believe ICDS frontline health workers and supervisors will be motivated to continue the improved processes with technical support.

The program also envisions building greater ownership within the community. It will therefore actively involve community groups and unpaid volunteers to help implement the next phase, with the aim of creating greater demand and accountability for public health services. Studies in similar contexts have shown that community groups and unpaid volunteers have contributed to improving child health and nutrition and have taken on roles similar to frontline health workers.^26^^–^^28^

We have yet to evaluate this next phase of the approach. We do not yet know the feasibility of transferring ownership of program activities to the government and community; however, findings from other countries suggest that CMAM programs run by NGOs during nutritional emergencies have been successfully handed over to governments for subsequent functioning.^29^^,^^30^ Findings from Bangladesh, Ethiopia, Malawi, and Niger suggest that handing over the CMAM activities to government health systems may be possible, and that it requires political commitment from the ministries, sustained resources, and general health system strengthening.^29^^–^^32^ However, a literature review of 33 studies of community-based malnutrition rehabilitation programs under routine health systems found that all successful programs had external support.^33^ In the Indian context specifically, training and supervision of frontline workers in government systems has been mentioned as an important hurdle.^34^ An assessment of the NGO–government partnership to enhance ICDS in northern India points to the challenge of scaling up a complex intervention through the existing health systems and staffing structures. The authors suggested the need for more rigorous and coordinated efforts with repeated reinforcement over longer periods of time.^35^

In this regard, we feel that an NGO–government partnership CMAM approach could provide technical support and additional resources to the government. Transferring ownership to the government may need to be gradual and in phases, with strong emphasis on ensuring that frontline health workers in the existing system are motivated, trained, and well-supervised—and have the resources available for effective day-to-day functioning.

### Limitations

The primary limitation of the qualitative study was that the views of the government partners were not included. Though stakeholders were questioned on the non-consumption of medical nutrition therapy, the reasons why it was not adopted widely were not explored specifically in this study. There is a need for further in-depth studies on understanding why the RUTF component was weak in our program.

The quantitative study had several limitations. First, the assessment of impact relies on cross-sectional endline data, which enables us to examine associations between variables but does not necessarily show causality. Second, the timing and length of the baseline survey in comparison areas was not optimal due to the practical constraints of conducting an evaluation within operational timelines. Due to limited time and financial resources, we used a shorter baseline survey in the comparison areas and could not conduct the data collection concurrently in both areas. We also did not conduct a comprehensive house listing in comparison areas as we had done in the intervention areas prior to the baseline. Our analysis of data from baseline to endline between intervention and comparison areas was therefore limited, and we could not provide a more rigorous explanation for the decline in wasting levels in the intervention areas using other statistical methods such as the difference-in-difference approach. There are seasonality implications on food availability and illness levels that affect wasting; however, due to timing and lack of data in the comparison areas at baseline, we were unable to control for differences that would likely affect dietary diversity and consumption of foods rich in vitamin A. We also do not have baseline data in the comparison areas on secondary outcomes, which limits our analysis in terms of showing the effects of intervention on infant and young child feeding practices, immunization coverage, vitamin A supplementation, and utilization of government health services.

Constraints at baseline limited our ability to provide a more rigorous explanation for the decline in wasting levels in the intervention areas.

Third, the intervention and comparison areas at endline were generally similar, with some differences particularly in the regional location of mothers' natal homes. We aimed to minimize these differences by controlling for a wide range of potential confounders. The endline data also indicated that no other organizations were working on child malnutrition in comparison areas and therefore no contamination was evident. Finally, the mixed results in secondary outcomes provided a limited explanation for the decline in wasting in intervention areas. Findings from other studies on the effectiveness of large-scale comprehensive nutrition programs is limited,^36^^,^^37^ typically focusing on primary outcomes without a deeper examination of the mechanisms for success or failure.^38^ Positive significant changes in secondary outcomes were observed in vitamin A supplementation, consumption of iron-rich foods, dietary diversity, and exclusive breastfeeding practices; however, overall levels of consumption remained low in intervention areas. We posit that the lower overall results for population-level behavior changes are due to the short duration of the intervention, as well as the focused counseling given to the target groups—wasted children and children under 6 months of age. Bringing down levels of malnutrition to achieve global targets and impacting the secondary outcomes will require more sustained efforts.

## CONCLUSION

To the best of our knowledge, this is one of the first evaluations of a large-scale urban CMAM program in India. While India bears the burden of the largest number of malnourished children in the world, evidence on both effectiveness and implementation processes of CMAM have largely been from African countries. With the growing interest in developing an in-country CMAM approach,^39^ concerns have been raised regarding the direct application of approaches from African countries (e.g., Ethiopia, Kenya, and Malawi) in India.^40^ Rural projects in Rajasthan, Bihar, and Madhya Pradesh^41^^–^^43^ have focused on the use of existing rural frontline health workers and public hospitals to piggyback on CMAM activities. Child malnutrition indicators in urban informal settlements and rural areas are equally distressing,^44^ yet there are few urban CMAM programs in India. Urbanization in India is increasing and urban community-based programs face specific challenges in migratory movement and the culturally heterogeneous nature of informal settlements. However, as was the case in the SNEHA program, crowded urban spaces also offer a unique advantage for frontline health workers to make frequent home visits with ease.^45^ The study was conducted as part of a large-scale program conducted in partnership with national and municipal government partners. This action-oriented research provides critical evidence for the kind of complex interventions required to achieve national and international objectives for improving child health.^46^^,^^47^

## Supplementary Material

17-00182-Jayaraman-Supplement.pdf

## Notes

**Funding:** Forbes Marshall Foundation and Epic Foundation funded the research.
