# Supplementary material for: Community-Based Management of Acute Malnutrition to Reduce Wasting in Urban Informal Settlements of Mumbai, India: A Mixed-Methods Evaluation
Source: Glob Health Sci Pract. 2018 Mar 21;6(1):103–27. doi: 10.9745/GHSP-D-17-00182 (PMC5878065; doi:10.9745/GHSP-D-17-00182)
Supplement: 17-00182-Jayaraman-Supplement.pdf [file 17-00182-Jayaraman-Supplement.pdf]

Jayaraman A, Shah More N, Waingankar A, et al. Community-based management of acute malnutrition to reduce wasting in urban informal settlements of Mumbai, India: a mixed-methods evaluation. *Glob Health Sci Pract*. 2018;6(1). https://doi.org/10.9745/GHSP-D-17-00182

Supplement: SNEHA CMAM PROGRAM

2011-2016

PRIMARY GOAL AND KEY RESULT AREAS OF THE CMAM PROGRAM

The primary goal of the Society for Nutrition, Education and Health Action (SNEHA) Community-based management of acute malnutrition (CMAM) program is to reduce the prevalence of acute malnutrition for children under three years of age by 25 percent. The SNEHA team identified additional Key Result Areas (KRAs) to measure progress towards this goal; they were selected on the basis of feasibility of change and strength of association with child undernutrition.

These KRAs include:

- Increase optimal breastfeeding practices among lactating mothers
  - Initiate breastfeeding within one hour of delivery
  - Exclusively breastfeed infants up to six months of age
  - Breastfeed continuously up to 15 months of age
- Improve complementary feeding practices in children 6 months to 3 years of age
  - Initiate complementary feeding in infants between 6-8 months of age
  - Infants and children receive appropriate quality and quantity of complementary feeds
- Reduce number of reported infections of illness (diarrhea)
- Improve coverage of beneficiaries under Integrated Child Development Services (ICDS)
  - Pregnant and lactating women receive health and nutrition advice from ICDS Frontline Workers (FWs)
  - Pregnant women consuming ICDS Take-home-rations (THR)
  - Children six months to 3 years of age consuming ICDS THR
- Improve Coverage of Vaccinations and Supplements
  - Children under age three are fully vaccinated
  - Children over nine months of age receive Vitamin A prophylaxis
  - Children over six months of age receive albendozole treatment

TEAM STRUCTURE

The overall core team structure as of March 2016 is presented in Figure 1:

Figure 1: Organogram of SNEHA CMAM Team (March 2016)

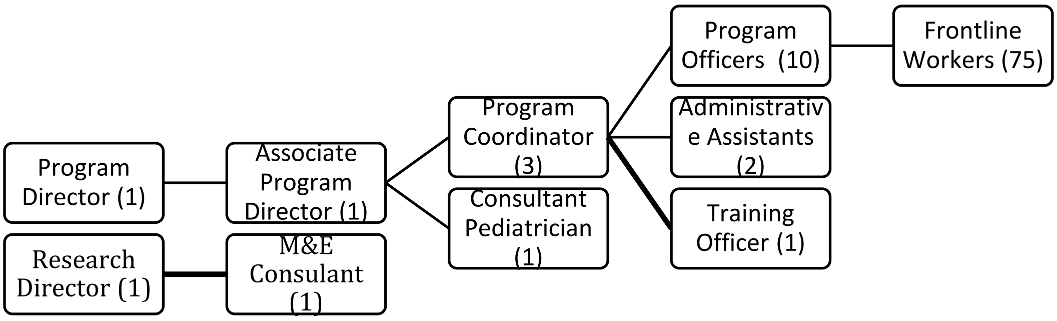

In addition to the core team there are also individuals who provide significant intellectual contribution to the program by providing advice and recommendations on a regular basis. This group comprises internal and external experts in the area of maternal and child health from various national and international organizations. During periods of evaluation and microplanning, temporary staff are hired for additional field level supervision and data entry.

Program Director and Associate Program Director

The SNEHA program is headed by the Child Health and Nutrition Program Director (PD) who drives the vision of the program by leading the team in achieving the objectives of the program. The PD works together with the Associate Program Director (APD) to manage the overall functioning of the team. The PD plays a large role in fundraising for the program and ensuring appropriate utilization of funds. Another important role for both the PD and APD is to develop stakeholder partnerships with ICDS, community health organizations, Municipal Corporation of Greater Mumbai (MCGM), and international organizations like UNICEF to collaborate for advocacy at the policy level. While the PD manages multiple programs, the APD works solely for the SNEHA CMAM intervention to manage the day-to-day operations and ensure the activities are completed on schedule.

Research Director & Monitoring and Evaluation Consultant

The SNEHA Research Director oversees the monitoring and evaluation (M&E) activities for the CMAM program. The M&E consultant develops the log-frame analysis, the design of the monitoring system and evaluation tools, and oversees the training of the intervention team on data collection processes. The consultant analyzes the data to generate monthly reports and inform the team on the progress of the KRAs. The M&E consultant works with the program coordinators to ensure the data is collected according to the established protocols and is accurate and complete.

Program Coordinators

The Program Coordinators (PCs) are responsible for the field-level planning and implementation of the activities of the project. They directly supervise a team of Program Officers (POs) to oversee training and capacity-building of team members, and communicate with other staff on documentation and M&E activities. They are required to work in close coordination with the relevant ICDS and MCGM staff to understand their functioning, and jointly plan and implement program activities. The PCs also oversee the activities of the consulting pediatrician providing medical screenings at the SNEHA community health camps.

Program Officers

The POs are responsible for the effective implementation of the program activities at the community level by monitoring and supervising FWs. They ensure: maximum coverage of beneficiaries in the intervention area; effective data collection; dissemination of information to the community; and conduct community outreach activities. The POs conduct qualitative and quantitative checks to ensure the quality of counselling, and data completeness and accuracy. Each PO is typically responsible for one administrative beat.

Frontline Workers

The FWs represent the biggest driving force of the organization and directly report to their respective PO. The FWs are key players in delivering the program activities as per program protocols and guidelines. Each beat is assigned 10 FWs and each FW is assigned three AWCs for their area of coverage.

Administrative Assistant

Administrative assistants are required to manage the daily office needs of the team. This includes managing the inventory and distribution of Ready-to-use-food (RUTF)to FWs.

Program Partnerships

The program works in close collaboration with communities and government systems. SNEHA improves linkages by strengthening the system of referrals between community members and public health systems. SNEHA's key partnerships in the CMAM program are with Integrated Child Development Services (ICDS) and the Municipal Corporation of Greater Mumbai (MCGM).

1. Integrated Child Development Services

ICDS, the largest national child welfare program functioning in India, launched in 1975 to tackle malnutrition and health problems in children below six years of age and their mothers. In Dharavi, Mumbai SNEHA operates in 10 ICDS administrative areas. From November 2011 through February 2014

SNEHA's work with ICDS was initiated with briefings with the ICDS Child Development Project Officer, ICDS Supervisors, and ICDS FWs from Dharavi. After program buy-in by the ICDS functionaries, a formal Memorandum of Understanding (MoU) was signed for a collaborative work project on an urban-CMAM model. The MoU established that SNEHA and ICDS would work together to weigh children, strengthen referral services, and provide counseling to caregivers of severely and moderately malnourished children.

2. MCGM Health Posts

An important collaboration that enables the program team to locally refer cases of illnesses, access immunizations and supplements, and promote ANC services is the presence of the MCGM health post. MCGM health posts carry out various health prevention and promotion activities including distribution of iron-folic acid tablets, vitamin A syrup, deworming drives, immunizations, and detection and treatment of TB, leprosy, and malaria. The health post covers a population of 50,000 and there is one available within each ICDS administrative beat. The SNEHA team coordinates with the health post staff to motivate and refer community members to attend relevant activities.

3. MCGM Tertiary Hospital—Lokmanya Tilak Municipal General Hospital (LTMGH)

The LTMGH hosts an essential component of the program at their Urban Health Center (UHC) in Dharavi – the Nutritional Rehabilitation and Research Center (NRRC). The NRRC encompasses a consultation room and production unit for RUTF cups called Medical Nutrition Therapy (MNT). MNT cups are used for in and outpatient treatment of severe malnutrition. Children with severe acute malnutrition (SAM) are referred by SNEHA's FWs to the NRRC, where their anthropometric information are recorded, an appetite test is administered and the doctor on-call examines the children for medical complications. The collaboration provides mutual benefit to both SNEHA and the NRRC Project of the hospital (see Table 1). The SNEHA program enables the NRRC to increase enrollment and coverage, and the NRRC Project provides SNEHA with medical services essential for diagnosis, referral, and treatment of SAM children. Following a SAM child's visit to the NRRC, children without complications are followed up at home by SNEHA FWs.

CMAM Interventions

Monthly Growth Monitoring

Each FW allocates approximately six days each month, two per AWC, to collect follow-up anthropometry for every child. Monthly weighing days are mutually scheduled with the ICDS FWs during the monthly planning meetings and the growth monitoring activity is held at the AWC. This aids in identifying normal children who may be faltering into malnutrition along with tracking the progress of children already screened as malnourished. Efforts are made to engage the mothers/caregivers in the process of monitoring the growth of the child. Caregivers are provided with WHO growth monitoring charts and taught how to track the growth status of her child on the chart. Caregivers are encouraged to bring the child herself for the weighing activity every month.

Referrals

Referrals to the public health systems are integral to the functioning of the program. Children can be referred to:

- MCGM Health Posts – Where all SAM and children with moderate acute malnutrition (MAM) are referred to for medical screening, vaccinations, and treatment of illnesses.
- LTMGH NRRC – SAM children with loss of appetite or medical complications are referred here for further screening and rehabilitation. Cases in need of in-patient care can receive treatment at LTMGH facilities.
- SNEHA Health Camps – Each month health camps are organized by the SNEHA program in every beat. These camps are organized in the community spaces and led by a consulting pediatrician trained in SAM management. Four to five health camps are organized in each beat every month.

Home Visits

Home-based counselling visits are planned and followed for each SAM, MAM, under-6-month child and pregnant woman. During the follow-up visits FWs visit homes to monitor the progress of the child and counsel the mother and family members on breastfeeding, complementary feeding, hygiene and sanitation, and illness treatment to improve the health of the child. Pregnant women are counseled on ANC, PNC, and breastfeeding practices such as initiation of breastfeeding within one hour of delivery and the importance of colostrum. Lactating mothers are counseled on breastfeeding, correct positioning, and the importance of exclusive breastfeeding. During the home visits, FWs collect information from the beneficiaries that help in monitoring the program's progress. The FWs closely monitor malnourished child and pregnant and lactating mothers during these follow-up visits. FWs can refer the child or mother to a health facility using referral slips and then follow up on the status of the referral on the next home visit.

MNT Provision

Along with home visits for counselling, FWs visit the homes of malnourished children to deliver MNT cups. MNT is a nutrient dense dietary supplement designed to treat severely malnourished children. It is fed to children 6-36 months of age who have an appetite and no medical complications. LTMGH, along with IIT Bombay, produces MNT using the WHO formula in a production unit located in the UHC, Dharavi.

Table 2: MNT		
**Nutrition Facts (Per 100g)**		**Ingredients**
**Protein**	16	Peanut, skimmed milk powder, powdered sugar, soybean oil, micronutrient mix and emulsifier.
**Carbohydrates**	44	
**Lipids**	34	
**Energy**	540 Kcal	

The intensity of the nutrition therapy varies according to the weight and nutritional status of the child. Children generally follow an 8-week protocol, consuming two doses daily. If the child has not reached the target weight (+15% of current weight) within two weeks, the child is re-assessed by a health camp or NRRC doctor. If a child has defaulted from the 8-week protocol or is irregularly consuming the MNT, the FW counsels the mother or primary caregiver on the importance of regular consumption of MNT. Tools have been developed and provided to caregivers to track MNT consumption at home. MNT will be discontinued for a week if the child has diarrhea and under the doctor's guidance it can be restarted after the child has recovered.

Community Advocacy

Bringing about positive behavior change through outreach activities in the community is another strategy of the program where larger groups of women and other community members are reached out to and messages are disseminated in creative ways. The women are involved in group activities that are fun and enjoyable such as educational games, celebrations of local festivals, and cooking demonstrations.

SNEHA Snakes and Ladders game: This is a modified version of the famous Snakes and Ladders board game that is enjoyed by all age groups. The original game has 100 squares on a board and is played by more than 2 players using a dice and tokens. Some of the squares on the board have ladders and snakes in between connecting two numbers that lead the player to a higher and lower number depending on whether the player lands on a square with a ladder or a snake respectively. For conducting such health and education activities like Snakes and Ladders or movie screenings, the community women are mobilized and invited to a location where the activity is organized. Each FW organizes such activities each month in their assigned areas. Sometimes such activities are done more than once to cover a larger number of women. Men and adolescent girls are also involved in such activities.

Meta Melavas: Rallies and mothers' group meetings called Mata Melavas are also organized at the community-level. For organizing a rally the SNEHA FWs of all the administrative areas get together, usually with the ICDS FWs, and walk in the alleyways calling out slogans and covering the entire area of each beat. The FWs also carry placards and banners with slogans written in various languages due to the diverse nature of the areas. Before organizing a rally permission from the respective police station is obtained. Because organizing a rally is an exhaustive exercise, it is done once in two to three months for each beat.

Capacity Building

Training is an ongoing process and an indispensable element of the program. The training is primarily carried out by the Training Officer and PCs. For specific topics external subject experts are invited. The experts also help train the trainers to carry out those trainings in future. Training is conducted SNEHA and ICDS FWs to build the capacity of the frontline staff and develop sound and consistent knowledge about malnutrition and the IYCF guidelines. Refresher trainings are also conducted for deeper understanding of the subject and reinforcement of the key messages that need to be delivered in the community.

Along with ensuring correct technical content, FWs receive substantive training on effective communication techniques. POs conduct spot checks in the field to assess the quality of the home visits which includes interpersonal skills like body language, appropriate introduction, appreciation of the caregiver, and active listening. POs are required to monitor each FW's counselling skills in the field every month. The POs give feedback to the FWs based on their observations and good performances are shared with the entire team during monthly meetings.

Training Modules

The following are the modules on which the training is provided to the SNEHA FWs and ICDS FWs. Some trainings such as the CommCare and SAM and MNT trainings are only provided to SNEHA FWs since ICDS FWs are not mandated to do those activities.

CommCare Training: This is a five-day training session which introduces the FWs to the use of the phone and the CommCare application that is used for data collection. The various monitoring tools used for data collection are also shared and explained to the FWs.

'I am a good mother': This module is the first in a series of modules on community-based management of malnutrition. It is developed in the belief that every mother does her best to bring up her child/ children. It intends to make the woman feel proud of her motherhood and motivates her to continue what she does well and at the same time adopt healthier child care practices to bring her child out of malnutrition.

Anthropometry and Grading: In this module, training is provided on taking accurate anthropometric measurements such as the height/length, weight, Mid-Upper Arm Circumference (MUAC), head circumference etc. The FWs are also trained on how to use the WHO tables to grade the children and assess the malnutrition status. This training is offered as classroom training as well as practical training in the field.

Malnutrition: The goal of the program and basic understanding about malnutrition and the indicators to assess malnutrition are covered in this training. This training relates the trainees to the local context and issues of malnutrition in the urban setting and the causes of malnutrition.

Balanced Diet: This module introduces the basic concepts of nutrition and a balanced diet. The various food groups and the importance of the nutrients and their functions are explained.

Breastfeeding: This is one of the most important modules which focuses on optimal breastfeeding practices and the importance of exclusive breastfeeding in the first six months after birth. The FWs are trained on various breastfeeding counseling techniques and correct positioning which they can demonstrate to the mother.

Complementary Feeding: The age of initiation of complementary feeding and its amount and frequency are explained in this module. During this training the FWs are trained on the use of standardized cups to explain the adequate quantity of feed to be given to the child at different age groups.

Immunization: This module encompasses the complete immunization schedule and the time and site of each vaccination. The main aim of this training is to convey the importance of complete immunization.

Common Childhood Illnesses: Key messages on the do and don'ts during common childhood illnesses are covered in this module.

Hygiene and Cleanliness: The differences between communicable and non-communicable diseases and the ways to prevent against communicable diseases are explained in this module. Diarrhea prevention, and the importance of hand washing and boiling drinking water is also covered in this module.

SAM and MNT: Because the FWs distribute MNT in the community they are briefed about what MNT is and its protocol. The use of MNT as a therapeutic food for the treatment of SAM is also explained to the FWs.

ANC and PNC: Guidelines of ANC/PNC and the danger signs of pregnancy are explained through this module. This module prepares the FWs to counsel the pregnant women and encourage them to register their names in a medical facility for ANC and take correct medical care after delivery. Key education on the importance of iron and folic acid, diet, and rest are included in this training.

Monitoring System

The Monitoring System was developed to periodically monitor the progress on key activities and outcomes as identified in the KRAs. The monitoring process involves collection of quantitative data, analyzing it, communicating results through regular reports, and taking corrective action on the basis of the findings. Quantitative information collected includes screening details, anthropometric data of children at each time of measurement, information collected during home visits on IYCF practices and other relevant health behaviors.

All community-level data is electronically collected by FWs with the use of Android smartphones. The monitoring forms for data collection were developed in a software application called CommCare (Dimagi, USA). The use of the CommCare platform enables data collection and storage on a web server called CommCareHQ using a standard mobile network. Monitoring forms were first created on paper and then programmed into the CommCare application. Once the form is completely filled by the FW, it is sent to the server over a cellular data network. All submissions made by the FWs can be viewed on the CommCareHQ website and downloaded from the server into Excel for reporting and analysis. CommCare is well suited for community-level work as the data can be entered by the FW offline if internet connectivity is unavailable. Every month the data that is collected by the FWs is extracted from the CommCare and analyzed. A monthly report is created.

Funding information

The SNEHA CMAM program raised funds through philanthropic foundations, corporate foundations, and individual philanthropists. Details of the program costs are presented in Table 2.

Table 2: SNEHA CMAM Project Expenses

**Budget Heads**	**Nov 2011 to Dec 2012**	**Jan 2013 to Dec 2013**	**Jan 2014 to Dec 2014**	**Jan 2015 to Dec 2015**	**Nov 2011 to Dec 2015**	
	**Year 1 Expenses**	**Year 2 Expenses**	**Year 3 Expenses**	**Year 4 Expenses**	**Total Expenses**	**% of Total Expenses**
Project personnel cost - salary cost of Associate Program Director, Program Coordinators, Program Officers, Field workers, Communication Manager, Training Officer, Data Team, Admin Team, Accounts Assistant and Pediatrician	46	85	117	112	**359**	**64%**
Project administration and overhead - Printing & Stationery, Project Office Rent, Communications cost comprising of telephone, internet data card etc., travel cost & staff professional development cost	18	25	36	30	**109**	**19%**
Project Direct Implementation Costs - Trainings, meetings, Monitoring & Evaluation, Events & Campaigns, ongoing expenses in field, IEC materials & training manuals	20	18	18	21	**78**	**14%**
Capital Expenses - Netbook, Furniture, Weight & Height Scales for Field Workers, Data collection devices etc.	6	5	2	1	**14**	**3%**
**Grand Total of Project expenses**	**90**	**133**	**173**	**163**	**560**	**100%**

(In INR Lakhs)
